# Reduced expression of *AtNUP62* nucleoporin gene affects auxin response in *Arabidopsis*

**DOI:** 10.1186/s12870-015-0695-y

**Published:** 2016-01-05

**Authors:** Martin Boeglin, Anja Thoe Fuglsang, Doan-Trung Luu, Hervé Sentenac, Isabelle Gaillard, Isabelle Chérel

**Affiliations:** Biochimie et Physiologie Moléculaire des Plantes, CNRS/INRA/SupAgro/UM2, 2 place Viala, 34060 Montpellier cedex, France; Present address: Plant and Environmental Sciences, Section for Transport Biology, University of Copenhagen, Thorvaldsensvej 40, 1871 Frederiksberg C, Denmark

**Keywords:** Nuclear pore complex, Nucleoporin, Auxin, Arabidopsis

## Abstract

**Background:**

The plant nuclear pore complex has strongly attracted the attention of the scientific community during the past few years, in particular because of its involvement in hormonal and pathogen/symbiotic signalling. In *Arabidopsis thaliana*, more than 30 nucleoporins have been identified, but only a few of them have been characterized. Among these, AtNUP160, AtNUP96, AtNUP58, and AtTPR have been reported to modulate auxin signalling, since corresponding mutants are suppressors of the auxin resistance conferred by the *axr1* (auxin-resistant) mutation. The present work is focused on AtNUP62, which is essential for embryo and plant development. This protein is one of the three nucleoporins (with AtNUP54 and AtNUP58) of the central channel of the nuclear pore complex.

**Results:**

*AtNUP62* promoter activity was detected in many organs, and particularly in the embryo sac, young germinating seedlings and at the adult stage in stipules of cauline leaves. The *atnup62-1* mutant, harbouring a T-DNA insertion in intron 5, was identified as a knock-down mutant. It displayed developmental phenotypes that suggested defects in auxin transport or responsiveness. *Atnup62* mutant plantlets were found to be hypersensitive to auxin, at the cotyledon and root levels. The phenotype of the *AtNUP62-GFP* overexpressing line further supported the existence of a link between AtNUP62 and auxin signalling. Furthermore, the *atnup62* mutation led to an increase in the activity of the *DR5* auxin-responsive promoter, and suppressed the auxin-resistant root growth and leaf serration phenotypes of the *axr1* mutant.

**Conclusion:**

AtNUP62 appears to be a major negative regulator of auxin signalling. Auxin hypersensitivity of the *atnup6*2 mutant, reminding that of *atnup58* (and not observed with other nucleoporin mutants), is in agreement with the reported interaction between AtNUP62 and AtNUP58 proteins, and suggests closely related functions. The effect of AtNUP62 on auxin signalling likely occurs in relation to scaffold proteins of the nuclear pore complex (AtNUP160, AtNUP96 and AtTPR).

**Electronic supplementary material:**

The online version of this article (doi:10.1186/s12870-015-0695-y) contains supplementary material, which is available to authorized users.

## Background

The nuclear pore complex (NPC) is a huge multiprotein complex, which controls exchanges of macromolecules (RNAs and proteins) between the nucleus and the cytosol in eukaryotes. It forms a doughnut-shaped, eight-fold symmetry structure composed of nucleoporins assembled in different kinds of complexes, forming a spoke/ring structure arranged around a central channel [1–3]. Whereas macromolecules readily diffuse through the NPC when their molecular weight is lower than ca. 30 kDa [4], they need to interact with nucleoporin-associated receptors (importins and exportins) to be carried from one side to the other when their molecular weight is larger than 40–50 kDa [4, 5]. The NPC is organized in interconnected subcomplexes with distinct functions. Some nucleoporins form the scaffold of the NPC structure, whereas FG nucleoporins, which display large domains containing multiple Phenylalanine-Glycine repeats, are involved in transport of macromolecules in the tunnel by binding to receptor-cargo complexes [5].

The plant NPC has recently attracted the interest of the scientific community due to the discovery of its contribution to several signalling pathways and, so far, 30 nucleoporins have been identified in *Arabidopsis thaliana* [6]. Plant nucleoporins are involved in cell responses to diverse hormonal signals as well as to biotic or abiotic environmental stimuli, such as auxin, symbiosis and pathogen attack [3, 7–9]. Suppressor of Auxin Resistance (*sar1* and *sar3*) Arabidopsis mutants, which were screened as suppressors of *axr1* (auxin-resistant 1), happened to be invalidated in nucleoporin genes (Arabidopsis *NUP160* and *NUP96/MOS3*, respectively) [10]. *Sar1 sar3* double mutants are deficient in mRNA export. Furthermore, regarding auxin signalling, the *sar3* and double *sar1 sar3* mutants display altered localization of AXR3/IAA17, which is an auxin response repressor from the AUX/IAA family. In these mutants, AXR3/IAA17 was not confined to the nucleus, as expected, but found throughout the cell, suggesting that AUX/IAA repressors are not properly imported or poorly retained inside the nucleus [10]. A nucleoporin of inner filaments of the nuclear basket, named AtTPR or NUA, is also involved in auxin signaling [11], and the *nua* mutant is deficient in mRNA export like *sar1* and *sar3* [11, 12]. Based on two-hybrid and genetic interactions, a functional relationship has also been suggested between AtNUP58 and hormonal (auxin/gibberellin) and light signalling [13].

This work is focused on the FG nucleoporin AtNUP62 from *Arabidopsis thaliana*, which is believed to be the orthologue of yeast Nsp1p and vertebrate Nup62 nucleoporins [6, 14]. It is located in the central channel of nuclear pores, together with AtNUP54 and AtNUP58, which are also FG nucleoporins [6, 15]. AtNUP62 is not homologous to other *Arabidopsis* FG nucleoporins, and it is the only one harbouring the Nsp1-C domain characteristic of yeast and vertebrate Nup62 [6]. Arabidopsis *AtNUP62* co-suppressors and mutants were reported to display a dwarf, early-flowering phenotype suggesting an important role in plant development [14, 15]. Overexpression of *AtNUP62* in tobacco leaves causes severe tissue decay in tobacco leaves [16]. A systematic search for embryo-defective mutants also identified two *atnup62* T-DNA insertion mutants [17]. In this study, we addressed the role of AtNUP62 from the point of view of auxin response.

## Results

### Variations of *AtNUP62* promoter expression

**Fig. 1:**
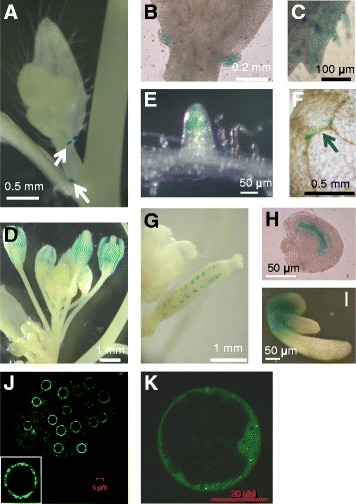
*AtNUP62* promoter activity and protein distribution*.* Tissue-specific activity of the *AtNUP62* promoter was investigated by histochemical analysis of *GUS* staining (blue color) in transgenic plants expressing *GUS* under control of *AtNUP62* promoter region. **a** Stipules at the basis of cauline leaves (arrows). **b** Enlarged view of stipules at the basis of a cauline leaf. **c** margin of a cauline leaf. **d** Inflorescence. **e** Root tip. **f** Cotyledon tip. The arrows indicates the localisation of GUS staining. **g** Young silique and **h** developing seed from this silique. **i** Young germinating seedling. **j** and **k**, Subcellular localization of AtNUP62::GFP fusion protein (confocal microscopy). The *AtNUP62::GFP* construct was expressed under control of the cauliflower mosaic virus 35S promoter, and the same construct was used for plant and protoplast transformation. **j** Root tip of a transgenic *35S::AtNUP62-GFP* 10-day old *Arabidopsis* plant. **k** Confocal microscopy analysis of AtNUP62::GFP signals in a transiently transformed *Arabidopsis* cultured cell protoplast

*AtNUP62* is not present on the *Arabidopsis* microarray chip (https://genevestigator.com/, Arabidopsis EFP browser: http://bar.utoronto.ca/efp/cgi-bin/efpWeb.cgi) and we have therefore not been able to obtain traditional microarray data. However, there are a few tilling array data (https://genevestigator.com/), indicating that the gene is expressed in flowers, seedlings and juvenile leaves, and to a lesser extent in adult leaves. In order to get more precisely the tissue-specific expression of the gene, transgenic plants expressing an *AtNUP62 promoter::GUS* fusion were created. The gene contains 8 small introns, all located in the last third of the ORF, at a minimum distance of 1.47 kb from the ATG. This distance, together with the negative IMEter scores of these introns (http://korflab.ucdavis.edu/cgi-bin/web-imeter.pl), make it unlikely that they might have a transcriptional activation function [18, 19]. The activity of the *GUS* gene under the control of *AtNUP62* promoter region allowed detecting a specific expression pattern (Fig. 1). Indeed, in adult plants, GUS staining was generally low, except in stipules at the base of cauline leaves, below flower buds (Fig. 1a and b). In other tissues, it was diffuse and preferably localized in developing organs, such as young leaves (Fig. 1c), flowers (Fig. 1d), and root tips (Fig. 1e). In young plantlets, a low-level expression could be detected in the area of veins at the cotyledon tip (Fig. 1f). At early stages, expression could be found in the embryo sac (Fig. 1g and h). The highest GUS activity was observed during germination (2 days after sowing, before the exit of the plantlet from the seed envelope), at the junction between the hypocotyl and cotyledons (Fig. 1i). At the subcellular level, the AtNUP62-GFP fusion protein was distributed at the nuclear periphery, as expected [14], with a clear punctuate pattern not previously observed (Fig. 1j). The same *AtNUP62-GFP* construct allowed detecting a broad fluorescence in the cytosol (Fig. 1k), which might be due to overexpression.

### Molecular characterization of the *atnup62-1* mutant

**Fig. 2:**
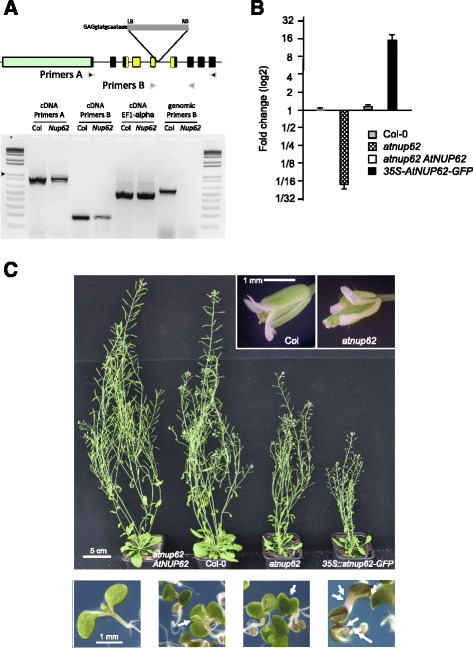
Analysis of the *atnup62* mutant. **a** Upper panel: Structure of the *AtNUP62* gene and position of the T-DNA insertion in the SALK_037337 (*atnup62-1*) mutant. Exons are represented by rectangles (9 exons) and introns by black lines. 5‘ and 3‘-UTR are thick red lines. The protein is composed of 739 amino acids. The ORF regions encoding the FG repeat, serine-rich domain (approximately 480 amino acids) and nsp1-C-like domain (amino acids 565 to 647) are figured in pale green and yellow, respectively. The T-DNA insertion site is indicated (LB and RB: left and right borders) with the sequence of the 15 bases just upstream of the insertion site, coming from our own sequencing of the junction region. The two pairs of primers (**a** and **b**) used for PCR are indicated. Lower panel: RT-PCR amplification of RNAs prepared from inflorescences of wild-type Col-0 (Col) and *atnup62-1* mutant (*nup*). Left lanes (amplification of cDNAs): after reverse transcription, PCR amplification was carried out using two couples of primers, **a** or **b**, for *AtNUP62*, or a couple of primers targeting the *EF1-α* gene. Right lanes (labelled Genomic DNA): amplifications of wild-type and mutant genomic DNA with primers B. Ladder: 1 kb plus from Promega, arrow at 1 kb. All amplified DNA fragments are present in the gel at a position consistent with in silico prediction. **b** Q-PCR analysis of *AtNUP62* expression in different genotypes. Results are expressed as fold changes compared to wild type. Values are means and standard errors of two biological samples (three technical replicates per sample). **c** Phenotype of *atnup62-1* mutant and complemented plants, at the adult stage (upper panels, zooms on flowers on top right) and on in vitro germinations on MS/2 medium (lower panels, 6 day-old seedlings). Lower panels, from left to right: wild type, plantlets with 3 cotyledons and 1 cotyledon from the *atnup62* mutant, plantlet with abnormal cotyledons from the *35S::AtNUP62*-GFP line, close to a plant (on the right) displaying a normal appearance. Arrows indicate abnormal plantlets. Cotyledons of complemented plants are similar to those of wild-type plants

The *AtNUP62* gene displays a specific organization with two completely different zones. The first zone is intronless, represents the initial two thirds of the ORF and encodes the FG repeat domain of the protein (Fig. 2a). Conversely, the second zone harbours multiple small introns. The encoded sequence displays many acidic and basic amino acids forming the Nsp1-C domain homologous to that found in the yeast Nsp1p nucleoporin.

We tried to isolate homozygous plant lines for two different alleles, SALK_071950 and SALK_037337, both displaying a T-DNA insertion in the 5^th^ intron. We were able to isolate viable homozygous seeds only from SALK_037337 (*atnup62-1*), in agreement with the previously observed lethal effect of the SALK_071950 (*atnup62-3*) mutation [15]. The third mutant, SAIL_127_F01 or *atnup62-2*, displaying a 53 amino acid deletion, has a less visible phenotype compared to *atnup62-1* [15] and was not used in this study. RT-PCR (Fig. 2a) and quantitative real-time PCR results (Fig. 2b) indicate that the *atnup62-1* mutant is not a bona fide knock-out plant but a mutant with reduced transcript levels. Sequencing PCR products (obtained with primers “B” in Fig. 2a) at the exon 5-exon 6-junction region revealed no difference in sequence between the wild type and the mutant (data not shown). Thus, in the mutant plant, the 5^th^ intron that contains the T-DNA can be spliced exactly as the 5^th^ intron of the wild type gene (at the position indicated by TAIR website, www.arabidopsis.org), albeit with decreased efficiency. Real-time quantitative PCR results (Fig. 2b) allowed concluding that the *atnup62* mutant displayed a strong reduction of the spliced transcript.

### Developmental defects of the *atnup62* T-DNA insertion mutant in reproductive organs and germinating seedlings

Previous analyses of a suppressive mutant [14] and two T-DNA insertion mutants [13, 15] revealed the same phenotypic defects. All plants impaired in *AtNUP62* function were small with reduced leaf blades and bolting occurred earlier than in the wild type.

We examined *atnup62-1* plants at different growth stages. In vitro-grown mutant seedlings (MS/2 medium) displayed small cotyledons (Fig. 2c, lower images) and frequent abnormalities such as cotyledon malformations, fused cotyledons and polycotyly (3 or even (rarely) 4 cotyledons; Fig. 2c and Table 1). This change in cotyledon number is exceptional in wild-type Col-0 plants (about 1 ‰). Cotyledons of mutant plantlets also became epinastic (Fig. 2c, second and third bottom pictures). In the greenhouse on compost, at the rosette and subsequent stages, mutant plants had smaller leaf blades than wild type plants and flowering occurred earlier, as described previously [14, 15]. Besides, other anomalies were also detected. Flowers were often abnormal and siliques much smaller than in wild type plants (Fig. 2c), with a longer desiccation time. We harvested 603 ± 15 mg of wild-type seeds per plant vs 270 ± 38 mg of mutant seeds from plants grown in parallel in individual pots. The *AtNUP62* cDNA under control of *AtNUP62* gene promoter complemented the germination and adult plant phenotypes (Fig. 2b and c) as well as the cotyledon phenotypes.

**Table 1:** Number of plants with cotyledon anomalies in the different genotypes

	Col-0	*atnup62*	*atnup62 AtNUP62*	*35S ::AtNUP62-GFP*
Normal cotyledons	418	360	407	180
2 abnormal cotyledons	5	24	1	14
3 cotyledons	0	52	0	3
1 cotyledon	0	5	0	0

Interestingly, the *35S::AtNUP62-GFP* transformed plant displayed a high level of *AtNUP62* transcript (Fig. 2b), a marked phenotype of reduced growth at the adult stage (Fig. 2c) and an intermediate phenotype in cotyledons (no or weak epinasty, but presence of a few plants with three cotyledons) (Table 1).

### The *atnup62* mutant and the *35S::AtNUP62-GFP* transformed plant are hypersensitive to auxin

**Fig. 3:**
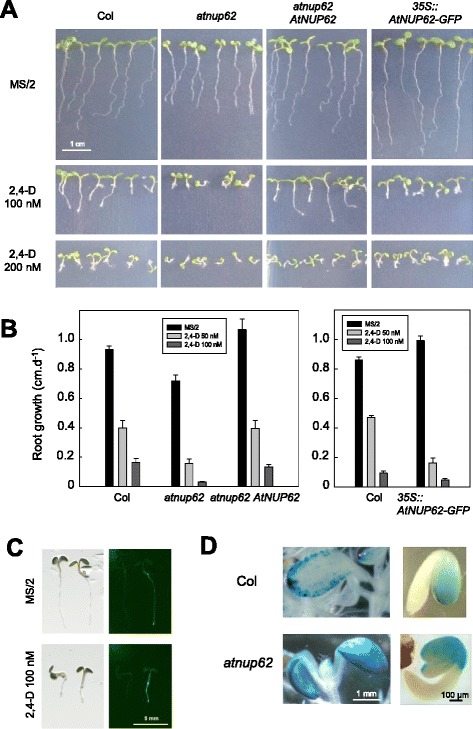
Auxin hypersensitivity of the different *AtNUP62* genotypes. **a** Growth on vertical plates (9 day-old plants) on different concentrations of 2,4-D. **b** Primary root growth measurements on plantlets grown on agar MS/2 medium containing different concentrations of 2,4-D (two experiments). Data are means ± SE. **c** Bright field images (left panels) and corresponding fluorescence images (right panels) in wild-type control plant (placed on the left) and a *35S::AtNUP62-GFP* plantlet, on MS/2 and 2,4-D-containing medium. Seedlings of comparable root size have been chosen. **d** GUS activity under the control of *DR5* auxin-responsive promoter in wild-type and *atnup62* mutant genetic background. Left, 11 day-old plantlets, right, 2.5 day-old seedlings

Auxin sensitivity of wild type and mutant plants was examined by growth on 2,4-dinitrophenoxyacetic acid (2,4-D), a stable auxin analogue. On 100 and 200 nM 2,4-D, aerial parts of wild type and complemented plants were moderately affected by the treatment, which mostly inhibited root growth (Fig. 3a).

Root growth on vertical agar plates was compared at the different concentrations of hormone. Whereas roots of Col, *atnup62*, complemented and *35S::AtNUP62-GFP* transgenic plants were all affected in the presence of 2,4-D, the effect was stronger in the mutant and in the *35S::AtNUP62-GFP* genotypes, whose residual primary root growth on 50 nM 2,4-D was only about 21 and 16 % of that on control medium, compared to about 40 % for the other two genotypes (Fig. 3b). A phenotype was also observed for the *atnup62* mutant plants in cotyledons. These organs, already reduced and epinastic in the absence of auxin analogue treatment, became tiny and severely distorted on 100 nM 2,4-D (Fig. 3a and Additional file 1: Figure S1). Despite their root phenotype similar to that of the mutant, the *35S::AtNUP62-GFP* plantlets did not display the phenotype of small epinastic cotyledons (Fig. 3a). This paradox can be explained by the distribution of the AtNUP62-GFP fusion protein, which appears to be almost absent in cotyledons both on MS/2 and 2,4-D-containing media (Fig. 3c).

The *DR5* synthetic promoter is commonly used to detect auxin activity [20]. In a wild type genetic background, *GUS* expression under the control of *DR5* can be detected in root tips, hydathodes and leaf margins ([20], and Fig. 3d). In cotyledons, intense blue staining was always restricted to leaf margins (Fig. 3d, left). Homozygous *atnup62* mutant seedlings harbouring the *DR5::GUS* construct, compared to isogenic *DR5::GUS* seedlings, displayed a higher GUS activity in cotyledons, in which the staining extended towards the centre of these organs (Fig. 3d, left). This suggested altered auxin activity in the *atnup62* plants. In young germinating seedlings, *DR5::GUS* activity was restricted to the cotyledons, the root tip and the inner edge of the hypocotyl alongside the cotyledons, in agreement with previous reports [21, 22]. The *atnup62* mutation resulted in an extension of the staining to the external upper part of the hypocotyl, where *AtNUP62* is expressed (Fig. 3d, right).

### AtNUP62 is a suppressor of auxin resistance conferred by the *axr1* mutation

**Fig. 4:**
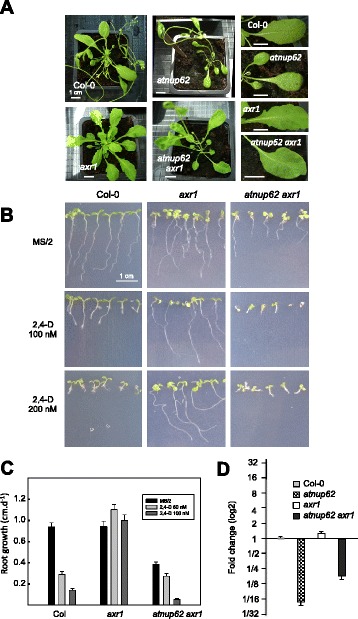
Effect of the *atnup62-1* mutation on the *axr1* mutant phenotype. **a** Plants grown in the greenhouse. The lower panels show the disappearance of the serrated margins of *axr1* plants in the *atnup62 axr1* double mutant. **b** In vitro-grown plantlets on MS/2 medium supplemented with different concentrations of 2,4-D. **c** Restoration of auxin sensitivity of primary root growth by the *atnup62-1* mutation in the *axr1* mutant. Data are means ± SE. **d** Q-PCR analysis of *AtNUP62* gene expression in *axr1* and the double *atnup62 axr1* mutant. Data are means ± SE of two biological samples (three technical replicates per sample)

The *AXR1* (auxin-resistant 1) gene encodes a subunit of the RUB-activating enzyme, necessary for the auxin-dependent degradation of AUX/IAA transcriptional repressors. *Axr1* mutants are resistant to auxin and also display a specific phenotype under standard growth conditions. This consists in a reduced height, defect in root gravitropism, abnormal inflorescences, low fertility [23], and serrated leaves [24]. In contrast to *axr1*, the *atnup62* mutation conferred sensitivity to auxin. We therefore created the *atnup62 axr1* double mutant. This mutant had lost the phenotype of leaf serrations (Fig. 4a). Leaves were also frequently embossed and misshaped, suggesting additional developmental problems that were not present in the parent lines (Fig. 4a). Most importantly, the auxin resistance of the *axr1* mutant was reversed by the *atnup62* mutation. Indeed, *axr1* plants had no apparent growth defect on 200 nM 2,4-D whereas double mutant plants were sensitive to the auxin analogue treatment (Fig. 4b, c and Additional file 1: Figure S1). The restoration of root sensitivity was partial on 50 nM 2,4-D but complete on 100 nM (Fig. 4c). Q-PCR analyses also revealed that the double mutant had a less reduced amount of *AtNUP62* transcript compared to the *atnup62* single mutant (Fig. 4d).

AXR1 mediates auxin response by activating the SCF E3 ubiquitin ligase complex that targets the AUX/IAA repressors of auxin response for ubiquitination and degradation [25]. In the auxin signalling cascade, this SCF complex is composed of SKP1/ASK1, the cullin CUL1 and the TIR1 F-box protein that specifically recognizes the AUX/IAA proteins to be ubiquitinated and degraded by the proteasome. A two-hybrid screening with AtNUP58, one of the other two FG nucleoporins of the central channel of the NPC, resulted in the identification of AtNUP62 (C-terminal Nsp1 domain), SKP1, and one of its homologues (SKP1B) [13]. SKP1 was found 8 times in that screening, always in frame with the activation domain of GAL4, from different positions in its sequence (from the 7^th^ to the 89^th^ amino acid). We therefore tested the hypothesis of an interaction between AtNUP62 and SKP1 (Additional file 2: Figure S2b), in both bait/prey combinations. AtNUP62 displayed a strong intrinsic transcriptional activity, leading to growth on medium lacking histidine (Additional file 2: Figure S2, right). The presence of SKP1 fused to the Gal4 activator domain (full-length clone obtained in a previous two-hybrid screening with another bait) did not increase the signal. In the reciprocal test, SKP1 displayed a weak transcriptional activity, which was not enhanced by AtNUP62 (Additional file 2: Figure S2).

## Discussion

### AtNUP62 expression and plant phenotype

*AtNUP62* is an essential gene, as demonstrated by the lethality of two out of four T-DNA insertions [15]. The developmental phenotype we observed for the *atnup62-1* mutant (anomalies in cotyledon shape and number, small siliques) is in accordance with the localization of *AtNUP62* promoter expression in the embryo sac and with the “embryo-defective” phenotype evidenced by the Seedgenes project [17]. Another mutant, *atnup62-3*, harbouring a T-DNA insertion in the same intron as *atnup62-1*, displays a more severe “embryo-defective” phenotype [17], leading to lethality ([15], this study). The presence of a residual ability to splice the fifth intron in *atnup62-1* might explain this phenotypic difference between the two mutants.

It also seems difficult to obtain plants that deeply overexpress the AtNUP62 protein. Zhao and Meier [14] obtained co-suppressors, but not overexpressors, by a strategy of plant transformation with a *35S::FLAG-AtNUP62* construct. Overexpression of *AtNUP62* by inoculation in tobacco leaves leads to very severe necrosis [16]. Despite the high level of *AtNUP62-GFP* transcript (Fig. 2b), the similitude of the phenotypes of *atnup62* and *35S::AtNUP62-GFP* lines indicates that the latter are loss-of-function rather than strong gain-of-function lines (Figs. 2c and 3), suggesting a dominant negative effect.

The localization of *AtNUP62* expression in the embryo sac and young seedlings (Fig. 1) is consistent with the embryo-defective phenotype, cotyledon and plantlet developmental defects. Interestingly, the *AtNUP62* promoter activity is concentrated in tissues that synthesize, accumulate or transport auxin. Cotyledon tips and embryos are specific expression sites for expression of *YUCCA* genes encoding enzymes in the auxin synthesis pathway [26]. In the root apex [27] and germinating seedlings [21, 22], the *AtNUP62::GUS* activity does not match with the *DR5-GUS* activity, but encompasses zones of active auxin transport (cotyledon veins, root tip and the apical hook of the young seedling). Stipules of rosette leaves have been reported, from the activity of the *DR5* auxin-responsive promoter, to accumulate free auxin [28], and have been postulated to regulate adjacent organ development [29]. Auxin is produced in flower organs, especially stamens [29], but a role of stipules of cauline leaves in flower development is not excluded.

### AtNUP62 and auxin signalling

It has been previously reported that some scaffold nucleoporins or NPC-associated proteins, AtNUP160/SAR1, AtNup96/SAR3 [10], AtNUP58 [13], and AtTPR [11], are involved in auxin responses. All mutants in these genes have in common the partial suppression of the *axr1* auxin resistance phenotype. The present results provide several lines of evidence supporting the hypothesis that AtNUP62 also plays a role in auxin signalling. Developmental defects, such as reduced size, small leaves [30], abnormal flowers [31] and early flowering [32] suggested that AtNUP62 could interfere with auxin distribution or signalling. The embryo and cotyledon phenotypes of the *atnup62* mutant are reminiscent of those of *mdr1* and *pin1* mutants impaired in polar auxin transport [33, 34] and *yucca* mutants impaired in auxin synthesis [26]. Cotyledon epinasty is one of the symptoms of auxin excess [26, 35], also displayed by the *atnup58* mutant [13], whereas the appearance of extra cotyledons is a sign of dysfunction in auxin distribution [36, 37]. Indeed, cotyledons, which are formed at the triangular stage of embryo development (between globular and heart stage), emerge from spots of auxin accumulation at the two side apexes of the triangle [34]. The *atnup62* mutation suppresses phenotypic traits of the *axr1* mutant, notably the auxin resistance, but also the typical serrated leaf phenotype that is auxin-dependent [24]. Finally, both *atnup62* mutant plants and the *35S::AtNUP62-GFP* plants are hypersensitive to auxin. This phenotype seems to be rather specific. Indeed, *atnup160/sar1* and *atnup96/sar3* do not display auxin hypersensitivity [15]. Among nucleoporin gene mutants, only *atnup58* was reported to be more sensitive than the wild type [13]. Interestingly, AtNUP58 is the only known nucleoporin that binds to AtNUP62, and reciprocally [13]. Hypersensitivity to auxin was also observed in mutant plants impaired in auxin membrane secretion [38] or control of AUX/IAA degradation [39].

An additional phenotype of the *atnup160*/*sar1* and *atnup96*/*sar3* mutants is the accumulation of mRNAs in the nucleus [10]. However the *atnup62-1* mutant does not accumulate nuclear mRNAs [15]. The same holds true for *atnup62-2* and mutants deficient for genes encoding other nucleoporins of the central channel of the NPC [15]. This suggests that accumulation of mRNAs in the nucleus and auxin-related phenotypes are independent phenomena.

Elucidation of the molecular basis of the relationship between AtNUP62 and auxin signalling will need further investigations. Essential genes are statistically prone to be highly connected in gene networks [40]. *AtNUP62* is notably connected to genes encoding nuclear pore proteins, and those involved in embryo development and protein ubiquitination (Aranet server, [41]). The decrease of *AtNUP62* transcript thus probably affects several networks. In order to address the molecular mechanisms affected in nucleoporin mutants, Parry [15] published a transcriptional analysis of *atnup160-4* and *atnup62-2*. Surprisingly, only a few genes displayed a more than twofold change in gene expression compared to the wild type. Among these, in a list of 18 genes up-regulated in both mutants, five were involved in nuclear transport and two (*SAUR9* and *ACS4*) were auxin-responsive. The identification of other targets would probably require tissue-specific RNAs or sampling at early stages (embryo development and germination).

We propose that AtNUP62 would act as a negative regulator of some auxin responses, like nucleoporins AtNUP160/SAR1 and AtNUP96/SAR3 [10]. The negative role of these two nucleoporins in auxin signalling has been ascribed to the fact that these proteins are involved in the retention of the transcriptional regulator AUX/IAA17 inside the nucleus. The suppression of the *axr1* phenotype by the *atnup62* mutation suggests that AtNUP62 also acts downstream of AXR1. The mechanisms that could underlie such a control needs to be better resolved, but two-hybrid interaction experiments with AtNUP58 [13] and the absence of evidence for a direct interaction of AtNUP62 with SKP1 (Additional file 2: Figure S2) suggest that AtNUP62 might modulate the activity or nuclear retention of SKP1 in part *via* its interaction with AtNUP58. This is corroborated by the similitude of plant phenotypes (cotyledon epinasty and root hypersensitivity to auxin). It is not excluded that AtNUP62 might also interfere with SKP1-like proteins (family of 21 members in *Arabidopsis*) [42], and/or some target components of the auxin signalling pathways, TIR/AFB receptors (6 members in Arabidopsis), AUX/IAA repressors (29 members) and ARF transcriptional factors (23 members) which form a complex combinatorial network, displaying specific expression patterns and involved in different types of responses [43].

## Conclusions

Our data provide a first explanation for the lethality of knock-out *atnup62* mutants, by highlighting its role in auxin-dependent development, especially at early stages. In the auxin signalling pathway, AtNUP62 would act downstream from AXR1. This also suggests that this plant FG nucleoporin, in close connexion with AtNUP58 and in relation to scaffold nuclear pore components (AtNUP160/SAR1, AtNUP96/S, and AtTPR), is able to take part in the control of other SKP1-dependent regulatory pathways, at the time and in the place where regulations are required.

## Methods

### *Arabidopsis* mutant lines

The *atnup62-1* and *atnup62-3* mutant lines (Col-0, SALK_037337 and SALK_071950) were obtained from the Nottingham Arabidopsis Stock Center [44]. Homozygotes were selected by PCR for insertion of T-DNA (primers at the T-DNA left border and in the *AtNUP62* gene) and for disruption of the *AtNUP62* gene (primers on both sides of the insertion site). The *axr1* mutant is *axr1-3* [23]. For the complementation of the *atnup62* mutant, *AtNUP62* cDNA was amplified by PCR using oligonucleotides 5′-TTGTAGGTCACCTCAAGACATCCAGTGCTTTGGAGCC-3′ and 5′-ACCCGCCATGGCGGGGTTTCCATTTGGTCAATCC-3′, digested with *Nco*I and *Bst*EII, and introduced into pCambia vector that had previously incorporated 1.8 kb upstream region from *AtNUP62* gene with *Nco*I site at its 3′ end.

### Plant growth conditions

Plants were grown in the greenhouse (8 h/16 h 21 °C /23 °C dark/light, light supplemented if necessary with sodium vapour lamps providing 150 μE.m^−2^.s^−1^) or in vitro in growth chamber (16 h-light photoperiod, 140 μM photons.m^−2^.s^−1^, 20 °C and 70 % humidity during both light and darkness) on half-strength Murashige and Skoog (MS/2) medium, supplemented when necessary with kanamycin (50 mg.L^−1^), hygromycin (30 mg.L^−1^) or 2,4-D.

### Localisation of *AtNUP62* promoter activity *in planta*

A 1.8 kb region upstream from the ATG of the *AtNUP62* gene (At2g45000) was amplified by PCR, using the 5′-GGTTACATTGTCGTGGTCGAGGTACG-3′ and 5′-CCCCGCCATGGCGGGTTATTGATTG-3′ primers and cloned into pBI-320X (*Sal* I/*Nco* I). The amplified promoter region was fused to the β-glucuronidase gene (*EcoR* I/*Sac* I fragment) and inserted into the binary vector pMOG406. *Arabidopsis thaliana* plants (Ws ecotype) were transformed using the floral dip method [45]. Transformants were selected on kanamycin, and homozygotes were recovered at the next generation. Beta-glucuronidase activity was detected according to [46].

### Localisation of AtNUP62 protein in protoplasts and plants by fusion with GFP

*AtNUP62* (At2g45000) cDNA was amplified with oligonucleotides 5′-CACCATGTCGGGGTTTCCATTTGGTC-3′ and 5′-AGACATCCAGTGCTTTGGAGCCA-3′ (ORF 5′ end and 3′ end without Stop codon), and cloned into pENTR/D-TOPO (Invitrogen). The cDNA sequence was introduced by LR recombination (Gateway LR clonase enzyme mix, Invitrogen) into pGWB5 (Tsuyoshi Nakagawa, Shimane University, Japan) in order to fuse GFP to the C-terminus of the AtNUP62 protein (resulting in pGWB5-*AtNUP62* plasmid used for plant transformation). Col-0 plants were transformed according to [45]. For transient expression in protoplasts, the *Hin*DIII/*Stu*I cassette (including the 35S promoter and GFP-coding sequences) was extracted from pGWB5-AtNUP62 and inserted into pGreen 0179, digested with *Hin*DIII and *Eco*RV. Fluorescence was detected under a Zeiss confocal microscope (LSM510 AX70 Zeiss, Göttingen, Germany). The excitation was obtained with a Beam splitter HFT 488, and the emitted radiations were selected with BP 505–530 nm filter.

### RT-PCR experiments

Inflorescences (including flower buds and young siliques) were pooled and ground in liquid nitrogen. RNA and genomic DNA were extracted from the same powder sample. RNA was extracted with RNeasy mini kit (Qiagen). Reverse transcription was achieved with 2.5 μg RNA using Superscript (Invitrogen). Two primer pairs, A and B, were used for PCR. (A): 5′-ACTCCGGCTAGCTCCGCTGCTAC-3′ and 5′-TTAGATCTTCAAGACATCCAGTGCTTTGGAGCC-3′ (STOP primer). (B): 5′-CTAGCTTGGAACGACAGCTGGA-3′ and 5′-TCTCTTTCTACTAGCTCAGACTG-3′. *EF1-α* was used as a control housekeeping gene, with primers5′-CCACCACTGGTGGTTTTGAGGCTGGTATC-3′ and 5′-CATTGAACCCAACGTTGTCACCTGGAAG-3′. To ensure that no wild-type sample contamination occurred in mutant samples during RNA extraction and subsequent steps, a completely independent repeat of RNA/DNA extractions and RT-/genomic DNA PCRs (primers B) was done and results were identical.

### Gene expression analysis

Total RNAs were extracted from plantlets grown on half-strength Murashige and Skoog (MS/2) medium for 8 days using the RNeasy Plant Minikit (Qiagen) and quantified by nanodrop after DNase I treatment (Invitrogen). First-strand cDNAs were synthesized with SuperScript III reverse transcriptase (Invitrogen), according to the manufacturer’s instructions. Oligonucleotides for gene-specific amplification were designed using PRIMER3 software (http://frodo.wi.mit.edu/primer3/). The primer pair AtNUP62-F (5′-GCAGAGTGGGATAAGCGGAT-3′) and reverse primer of pair B (5′- TCTCTTTCTACTAGCTCAGACTG-3′) spans intron IV and V, according to *AtNUP62* gene structure (Fig. 2), the 5^th^ intron containing the T-DNA insertion (Fig. 2a). For normalization, the *PDF2* reference gene (At1g13320) was selected on the basis of its expression stability in roots and leaves under our conditions (primers *PDF2-F* (5′-TAACGTGGCCAAAATGATGC-3′*)* and *PDF2-R* (5′-GTTCTCCACAACCGCTTGGT-3′)). PCR reactions were performed on a LightCycler 480 (ROCHE Applied Science) in triplicate with two independent biological samples. Absence of genomic DNA and primer dimers was confirmed by analysis of minus-RT and water control samples, and by examination of melting curves. Baseline data were collected between cycles 3 and 15. All amplification plots were analysed with an Rn (normalized reporter) threshold of 0.2 to obtain CT values. Data were analyzed using Roche LightCycler software and to derive relative expression levels, the comparative CT method (DDCT) was used as described in Cuéllar et al. [47].

### Selection of *atnup62* mutant plants harbouring the *DR5::GUS* construct

*Atnup62-1* homozygous mutant plants were crossed with plants homozygous for the *DR5::GUS* insertion [20]. The presence of SALK T-DNA insertion in *AtNUP62* gene and *DR5::GUS* integration were confirmed respectively by PCR and ß-glucuronidase activity, and homozygotes were selected at the next generation by checking the absence of *AtNUP62* PCR product and the presence of GUS activity in the progeny (100 % of GUS positive plantlets).

### Two-hybrid experiments

A partial *AtNUP62* cDNA encoding the NSP1 C-terminal domain (from S266 to the end of the polypeptide sequence) and the full-length *SKP1* cDNA were obtained by a two-hybrid screening of the FL4000AB cDNA library (Clontech). The full-length *AtNUP62* cDNA clone was obtained by PCR and inserted into pGBT9 [48] and pGAD10 (Clontech). The *Bgl*II fragment of the library *SKP1* cDNA clone was inserted into pGBT9 previously digested with *Bam*H1. Yeast transformation (AH109 strain) was performed according to [49]. Yeast transformants were grown in liquid medium lacking tryptophan and leucine, cells were washed with water, and threefold serial dilutions (first dilution corresponding to an OD of 0.066 at 600 nm) were dropped on medium lacking tryptophan, leucine and histidine [50].

### Availability of supporting data

All the supporting data are included as additional files.

## Additional files

Additional file 1: Figure S1. Auxin sensitivity of the *atnup62* and *atnup62 axr1* mutants (top views). (PDF 466 kb) Additional file 2: Figure S2. Absence of AtNUP62-SKP1 interaction in two-hybrid tests. Scheme of the transcriptional auxin signalling pathway and two-hybrid tests. (PDF 228 kb)

## Abbreviations

NPC: Nuclear pore complex
2,4-D: 2,4-Dinitrophenoxyacetic acid
