# Supplementary material for: Reduced expression of AtNUP62 nucleoporin gene affects auxin response in Arabidopsis
Source: BMC Plant Biol. 2016 Jan 5;16:2. doi: 10.1186/s12870-015-0695-y (PMC4700657; doi:10.1186/s12870-015-0695-y)
Supplement: Additional file 1: Figure S1. — Auxin sensitivity of the atnup62 and atnup62 axr1 mutants (top views). (PDF 466 kb) [file 12870_2015_695_MOESM1_ESM.pdf]

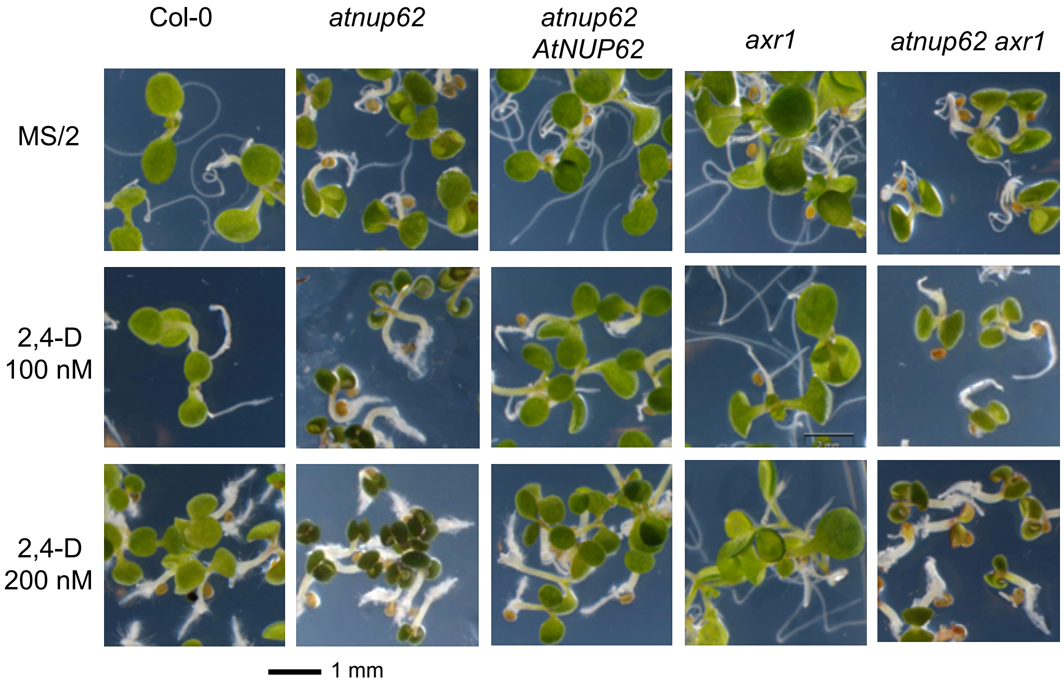

Figure S1. Auxin sensitivity of the *atnup62* and *atnup62 axr1* mutants (top views). Effect of 2,4-D on seedling growth, 6 day-old plantlets.
