# Supplementary material for: Reduced expression of AtNUP62 nucleoporin gene affects auxin response in Arabidopsis
Source: BMC Plant Biol. 2016 Jan 5;16:2. doi: 10.1186/s12870-015-0695-y (PMC4700657; doi:10.1186/s12870-015-0695-y)
Supplement: Additional file 2: Figure S2. — Absence of AtNUP62-SKP1 interaction in two-hybrid tests. Scheme of the transcriptional auxin signalling pathway and two-hybrid tests. (PDF 228 kb) [file 12870_2015_695_MOESM2_ESM.pdf]

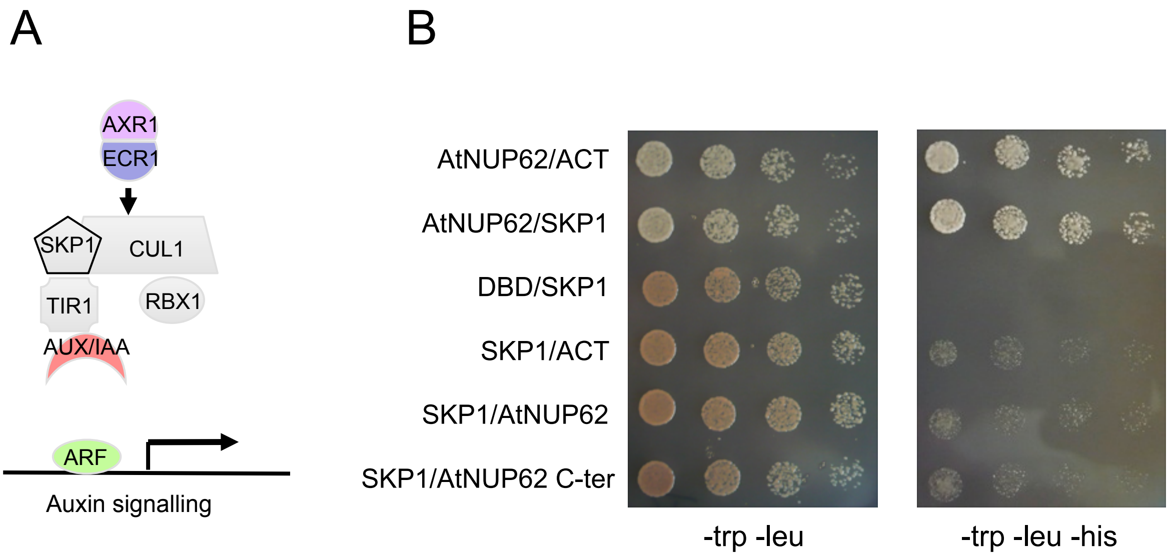

Figure S2. Absence of AtNUP62-SKP1 interaction in two-hybrid tests. **(a)** Classical model of transcriptional auxin signalling. **(b)** Two-hybrid tests (bait/prey) between AtNUP62 and SKP1. Left, growth of yeast transformants on medium that does non select for *HIS3* reporter gene activation. Right, growth on selective (minus histidine) medium. ACT: activator domain alone (empty prey vector), DBD: DNA-binding domain alone (bait vector), AtNUP62 C-ter: clone obtained from a screening of the cDNA library (encoding AtNUP62 protein from S266).
